# Public Awareness and Attitude Regarding the Symptoms of Heart Attacks

**DOI:** 10.5334/gh.1492

**Published:** 2025-12-02

**Authors:** Alya A. Aljubran, Jumanah A. Almubarak, Kawther H. Alawad, Wejdan A. Alolaywi, Rabab A. Almarzooq, Hussain N. Alali, Mohammed S. Alsaad, Mustafa S. Albagshi, Zainab Amjad, Eman Elsheikh

**Affiliations:** 1 College of Medicine, King Faisal University, Al-Ahsa, Saudi Arabia; 2 Internal Medicine Department, College of Medicine, King Faisal University, Al-Ahsa, Saudi Arabia; 3 Cardiovascular Department, College of Medicine, Tanta University Hospital, Tanta, Egypt

**Keywords:** heart attack, awareness, prevalence, risk factors, Saudi Arabia, public health, emergency response

## Abstract

**Objective:**

To assess the heart attack (HA) knowledge, awareness, and attitude among adults in Al-Hasa, Saudi Arabia, and to identify gaps in understanding that could hinder prompt medical intervention.

**Methods:**

A descriptive cross-sectional study was conducted using a self-administered questionnaire distributed via Google Forms from January to March 2024. Statistical analysis was performed using IBM SPSS, Version 29, to evaluate associations between demographics and HA awareness.

**Results:**

Participants demonstrated moderate awareness of non-classical HA symptoms such as slurred speech (relative importance index (RII) = 72.88%) and dizziness (66.35%), whereas critical symptoms such as chest pain (47.8%) and shortness of breath (47.25%) were among the least recognized. This suggests a concerning gap in knowledge of the most urgent indicators of HA. Respondents showed higher awareness of non-modifiable risk factors such as family history (RII = 70.99%) and high cholesterol (63.92%) compared to modifiable lifestyle-related risks. Smoking (43.71%) and obesity (43.08%) ranked lowest in awareness, indicating insufficient recognition of preventable contributors to cardiovascular disease. Participants exhibited a high level of hesitation in seeking immediate medical attention during a suspected HA. Social embarrassment (RII = 67.36%) and concerns about healthcare costs (66.08%) were the primary reasons cited for delay. Alarmingly, the belief that one should wait to be ‘very sure’ before going to the hospital was common (RII = 59.01%), whereas the urgency of symptoms such as persistent chest pain was undervalued (RII = 31.18%). Significant differences in symptom recognition were observed across age groups (e.g., *P* = 0.001 for jaw/neck/back pain), education levels (e.g., *P* = 0.028 for pain in arms/shoulders), and marital status (e.g., *P* = 0.002 for several symptoms). No significant gender-based differences were found.

**Conclusion:**

Al-Hasa population showed good knowledge and awareness of HA symptoms and risk factors; however, significant gaps exist in recognizing less common symptoms and emergency procedures. Poor attitude was shown toward HA seeking medical care. Concerns about cost, embarrassment, and suspicion in the severity of the symptoms appeared to be barriers to seeking timely care.

## Introduction

A heart attack (HA), or myocardial infarction (MI), is a major clinical consequence of cardiovascular diseases (CVDs), resulting primarily from sudden blockage of coronary arteries. CVDs account for around 40% of deaths in the European Union (1) and 35% in the United States (2). The most common clinical manifestation of CVDs is acute coronary syndrome (ACS), a term that encompasses a spectrum of conditions caused by an abrupt reduction in coronary blood flow, usually due to acute thrombotic obstruction following plaque rupture or erosion. Risk factors such as diabetes, hypertension, dyslipidemia, obesity, smoking, undesirable dietary habits, and physical inactivity are all predictors of ACS (3).

ACS remains a major issue in the Kingdom of Saudi Arabia, contributing to a cardiovascular mortality rate of approximately 454.5 per 100,000 population as of 2022 (4). Dyslipidemia and hypertension are the predominant risk factors. According to a recent study, 15.6% of individuals have one cardiovascular risk factor, 24% have two, 16.9% have three, and 17.6% have four risk factors (5). According to a study conducted in Saudi Arabia, more than 40% of ST-elevation MI (STEMI) patients failed to reach a 90-minute door-to-balloon time, with women having a worse chance of meeting this standard. The in-hospital death rate was 4% (6).

Understanding the first symptoms of ACS, including MI, is crucial for saving lives through quick action by the patient or others. Alfasfos et al.’s (7) survey of Jordanians showed that the most common sign of ACS is chest pain, while arm numbness and shortness of breath are also common; however, many respondents ignored such symptoms as abdominal discomfort and heartburn/indigestion. In another study carried out on Lebanese participants, Noureddine et al. showed that the majority of the participants (>85%) named the classic signs of MI, including chest discomfort and sweating, while fewer recognized dyspnea (66%), arm pain (62%), and nausea/vomiting (52%). Less than half of the patients described atypical symptoms, such as heartburn and jaw pain (8).

According to a study by Fang et al. (9) in the United States, the adjusted percentage of people aware of all five common HA symptoms (shortness of breath, chest pain, arm or shoulder pain, weakness or lightheadedness, and jaw, neck, or back pain) increased from 39.6% in 2008 to 50.0% in 2014 and 50.2% in 2017. Similar studies have been conducted in Bangalore, India, and in Beijing and Shanghai, China. Only 60% of people in all cities recognized chest pain or discomfort as a sign of acute MI (AMI), according to the research. Twenty-one percent of participants in Bangalore were unable to recognize any symptoms (10).

Women often had longer delays in seeking treatment due to misattributed symptoms, social factors, or healthcare access issues than men; Lichtman et al. (11) found that younger women (<55 years old) had longer delays than older patients. Due to a number of factors, including (1) considerable variation in the type and duration of prodromal symptoms; (2) patients’ misestimation of their own risk of heart disease and their tendency to attribute symptoms to non-cardiac causes; (3) competing and conflicting priorities influencing decisions to seek medical attention; (4) the healthcare system’s inconsistent response to young women experiencing MI, which resulted in delays in evaluation and diagnosis; and (5) participants’ irregular engagement with primary care, including CVD prevention, women under 55 years old who experienced MI had a higher in-hospital mortality rate compared to men of the same age group, with an adjusted odds ratio (OR) of 1.5 (95% confidence interval (CI): 1.3–1.7), indicating a 50% higher risk of death (11).

The incidence of MI-related complications has increased due to delays in seeking medical care. Several factors contribute to these delays, including a lack of awareness of symptoms and their consequences, misinterpreting symptoms as less serious conditions, competing personal priorities, and reluctance to inconvenience others. Enhancing public awareness of MI symptoms could significantly reduce the time to treatment and, in turn, substantially lower the one-year mortality rate. Additionally, improving public understanding of thrombolytic therapy is a key element in raising awareness and encouraging timely response to MI. Despite medical advances over the past two decades, public knowledge and understanding of AMI remain insufficient, which is a concerning issue (9). Thus, this study aims to assess HA awareness among adults in Al-Hasa, Saudi Arabia, and identify knowledge gaps that may delay medical responses. Al-Hasa, with over 1.3 million residents across urban and rural areas, was selected for its demographic diversity and high prevalence of cardiovascular risk factors, making it a relevant setting for public health research.

## Methods

### Study design and sample

This was a descriptive cross-sectional study conducted in Al-Hasa, Saudi Arabia, between January and March 2024. The target population consisted of Arabic-speaking adults aged 18 years or older residing in Al-Hasa. At the time of data collection, Al-Hasa had an estimated population of approximately 1.3 million residents, according to the 2023 national census. Healthcare professionals, academics, and students were excluded from the study to minimize bias from individuals with advanced medical knowledge that may not reflect the general public.

The minimum required sample size for this study was 385 participants, determined using the Raosoft sample size calculator. The calculation was based on a 5% margin of error, 95% confidence level, and an assumed 50% response distribution, which aligns with a moderate effect size (Cohen’s *d* ≈ 0.5), a common standard in population health research when prior effect size estimates are unavailable. Additionally, a 10% non-response rate was considered to ensure adequate power and representativeness of the sample.

For data collection, a non-probability convenience sampling technique was used from January to March 2024. Data were collected through self-administered questionnaires using Google Forms.

The survey was disseminated via local social media groups and community WhatsApp channels. It took approximately 5–7 minutes to complete. Google Forms was set to require login and to limit one response per participant to avoid duplicates.

The questionnaire was developed based on validated items from prior literature and then subjected to content validation by three experts in cardiology and public health from King Faisal University. Each expert independently reviewed the questionnaire for relevance, clarity, and comprehensiveness.

Items were rated using a 4-point relevance scale (1 = not relevant, 2 = somewhat relevant, 3 = quite relevant, and 4 = highly relevant). The content validity index (CVI) was calculated for each item, and those scoring below 0.78 were either revised or removed. Consensus was achieved through two iterative review rounds until all items reached expert agreement (I-CVI ≥ 0.80). This process ensured content validity, clarity, and contextual relevance to the Saudi population (12).

According to the Communications and Information Technology Commission (CITC), internet penetration in the Kingdom of Saudi Arabia, including the Al-Hasa region, exceeded 96% as of 2023. This widespread digital connectivity supports the feasibility of using online platforms for survey distribution across a broad demographic spectrum. In Al-Hasa, both urban and semi-urban areas have high smartphone usage and access to internet services, making online data collection via Google Forms an effective and inclusive approach for reaching the adult population.

### Measures

The questionnaire was divided into three parts. The first part collected socio-demographic data such as age, gender, education level, and marital and employment status. The second part assessed the participants’ knowledge of both risk factors and symptoms of HA, including typical and atypical symptoms. The third part explored the participants’ attitudes toward seeking medical care if they witnessed or suspected that someone was having an HA. Furthermore, it examined various factors that could influence an individual’s decision to seek help, such as cultural beliefs, level of certainty, and emotional and financial constraints.

For the purposes of this study, we distinguish between three related but distinct constructs: awareness, knowledge, and attitude. Awareness refers to the participants’ recognition of the existence or importance of HA symptoms and risk factors, regardless of depth of understanding. Knowledge refers to the participants’ accurate understanding of specific symptoms and risk factors, as measured by correct identification of clinical signs and modifiable contributors. Attitude refers to participants’ perceptions, beliefs, and behavioral intentions toward seeking medical care when faced with suspected HA symptoms. These definitions were used to structure the questionnaire and interpret findings across the three domains of assessment.

### Data analysis

Data analysis was conducted using IBM SPSS, Version 29, and Microsoft Excel. The statistical analysis in this study involved several key methods.

Reliability testing: Internal consistency was evaluated using Cronbach’s alpha across the three primary domains of the questionnaire. The values were 0.796 for the symptom’s domain, 0.781 for risk factors, and 0.801 for attitudes toward seeking medical care, all exceeding the acceptable threshold of 0.70, indicating good internal reliability across the items.

Validity testing: Construct validity was assessed through item-total correlation analysis. Each item within the symptom’s domain showed a significant positive correlation ranging from 0.410 to 0.775, supporting their contribution to measuring the intended construct. Similar correlation patterns were observed across the other domains, affirming overall content and construct validity.

Descriptive statistics: Mean, standard deviation, and relative importance index (RII) were computed for awareness of symptoms and risk factors. The RII was calculated as RII = (mean/3) × 100. Although responses were obtained on a 4-point Likert scale (1,2,3,4), the denominator ‘3’ was used to normalize the results to a 0–100 scale, facilitating comparison and interpretation across items. RII values were then categorized as low, medium, or high to indicate the level of awareness or agreement for each symptom or risk factor. This analysis highlighted areas where awareness was lower, such as for sudden chest pain or shortness of breath, indicating a potential need for targeted awareness campaigns.

Inferential statistics: Chi-square tests were used to examine relationships between demographic variables and awareness of HA symptoms. Chi-square tests assessed associations across full categorical variables, not between individual categories unless specified. Significant differences in awareness were observed based on variables such as age, education level, marital and employment status, as shown by *P* values < 0.05. For instance, awareness of ‘sudden pain in jaw, neck, or back’ was significantly different across age groups.

This combination of reliability, validity, descriptive, and inferential statistical techniques provided a comprehensive analysis of the survey data, highlighting both the strengths and gaps in public awareness and attitudes toward HA symptoms and risk factors.

### Ethical considerations

The protocols for this study were approved by King Faisal University’s Medical Research Centre (2024-MAR-ETHICS2053). Consent was obtained prior to completing the questionnaire, which was administered anonymously. All data would be kept confidential and utilized strictly for this study.

## Results

### Reliability and validity test

Reliability and validity data are provided in Supplementary Tables S1 and S2.

Cronbach’s alpha values across all domains ranged from 0.763 to 0.824, indicating acceptable to strong internal consistency (see Supplementary Table S1). Validity was established through significant positive item correlations within each domain, supporting the construct validity of the instrument (see Supplementary Table S2).

Table 1 presents a diverse sample of 424 participants in terms of age, gender, education, marital and employment status, and health conditions. The majority of respondents were aged between 18 and 55 years, with a near-even split between males and females. Educational attainment was relatively high, with most participants holding at least a high school diploma and over half possessing a bachelor’s degree or higher. Most participants were married and employed, though students, housewives, and retirees were also represented. While the majority reported no pre-existing health conditions, hypertension and diabetes were the most commonly reported chronic illnesses among those who did.

**Table 1:** Demographic characteristics of study participants.


CATEGORY	FREQUENCY	%

Age, years		

18–25	99	23.3

26–35	88	20.8

36–45	102	24.1

46–55	93	21.9

≥65	42	9.9

Sex		

Female	217	51.2

Male	207	48.8

Education level		

Illiterate	7	1.7

Primary school	4	0.9

Middle school	13	3.1

High school	109	25.7

Diploma	51	12

Bachelor’s degree	214	50.5

Postgraduate	26	6.1

Marital status		

Single	98	23.1

Married	308	72.6

Divorced	11	2.6

Widow	7	1.7

Employment status		

Employed	181	42.7

Unemployed	38	9

Student	86	20.3

Housewife	71	16.7

Retired	48	11.3

Pre-existing conditions		

Hypertension	60	14.2

DM	46	10.8

Dyslipidemia	29	6.8

Heart diseases	15	3.5

Stroke	2	0.5

None	305	71.9


**Figure 1:**
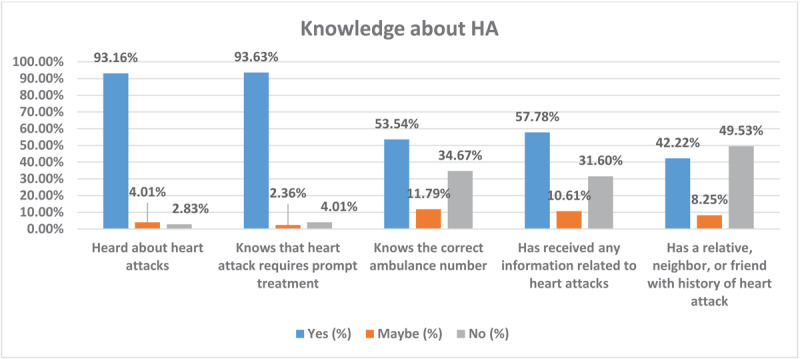
Participants’ knowledge about heart attacks (HAs). Bars show the percentage of respondents who answered each knowledge item affirmatively (e.g., 96% had heard about heart attacks, 93.2% knew that prompt treatment is required). Font style and label size are standardized across all figures for consistency.

Figure 1 shows 96% of the respondents had heard about HA, and 93.2% were aware that HA requires prompt treatment. However, only 53.5% were able to identify the correct emergency ambulance number, and only 57.8% received information related to HA.

Table 2 shows the source of information related to HA. The majority (63.29%) of respondents obtained it from the internet and social media, followed by healthcare professionals (15.9%), television (12.3%), and books (5.31%).

**Table 2:** Source of information about HA among the study participants.


SOURCE OF INFORMATION	FREQUENCY	PERCENT

TV	51	12.32

Internet and social media	262	63.29

Books	22	5.31

Healthcare professionals	66	15.94

Others	13	3.14

Total	414	100


Abbreviation: HA, heart attack.

Table 3 provides a detailed analysis of respondents’ awareness of HA symptoms and risk factors.

**Table 3:** Awareness of common and uncommon symptoms and risk factors of heart attacks.


FIRST DOMAIN: THE AWARENESS OF COMMON AND UNCOMMON SYMPTOMS OF HEART ATTACKS					

SYMPTOMS	M	SD	RII	LA	R

Sudden pain in jaw, neck, or back	1.9575	0.83544	65.25	Medium	4

Weakness or dizziness	1.9906	0.91992	66.35333	Medium	2

Sudden pain in the chest	1.434	0.80211	47.8	Low	9

Sudden disturbance of vision in one or both eyes	1.9599	0.91264	65.33	Medium	3

Sudden pain in arms or shoulders	1.9222	0.88917	64.07333	Medium	5

Palpitation	1.4175	0.79167	47.25	Low	10

Sudden shortness of breath	1.5071	0.84212	50.23667	Low	8

Stomach pain or abdominal pain	2.1863	0.71174	72.87667	Medium	1

Slurred speech	1.8373	0.9015	61.24333	Medium	7

Numbness or tingling in the hand	1.8561	0.90209	61.87	Medium	6

**SECOND DOMAIN: AWARENESS OF VARIOUS HEART ATTACK RISK FACTORS**					

**RISK FACTORS**	**M**	**SD**	**RII**	**LA**	**R**

Smoking	1.3113	0.696	43.71	Low	10

Obesity	1.2925	0.68371	43.08333	Low	11

Diabetes	1.8443	0.90927	61.47667	Medium	4

Lack of exercise	1.7995	0.92504	59.98333	Medium	5

Unhealthy diet	1.9316	0.92894	64.38667	Medium	2

Stress	1.7146	0.89979	57.15333	Medium	7

High blood pressure	1.4363	0.80524	47.87667	Low	9

High levels of cholesterol	1.9175	0.87263	63.91667	Medium	3

Genetics	1.5802	0.87421	52.67333	Low	8

Family history	2.1297	0.79874	70.99	Medium	1

Past history of HA	1.7311	0.90878	57.70333	Medium	6


Abbreviations: HA, heart attack; LA, level of agreement; M, mean; R, rank; RII, relative importance index ((mean/3) × 100%); SD, standard deviation.

The first domain assessed participants’ awareness of both common and uncommon HA symptoms. Among all symptoms, stomach or abdominal pain had the highest recognition score (RII = 72.88, medium level), suggesting it was the most commonly identified by participants. Other symptoms with medium-level awareness included weakness or dizziness (RII = 66.35), sudden vision disturbances (RII = 65.33), jaw, neck, or back pain (RII = 65.25), and pain in the arms or shoulders (RII = 64.07).

In contrast, several critical hallmark symptoms of MI—specifically palpitations (RII = 47.25), sudden chest pain (RII = 47.80), and sudden shortness of breath (RII = 50.24)—received low awareness scores, ranking among the bottom of the symptom list. These symptoms are well-established clinical indicators of HA and are essential for prompt identification and response. The relatively low recognition may be attributed to misattribution to non-cardiac conditions, such as anxiety, fatigue, gastrointestinal discomfort, or stress. In some cases, cultural beliefs and limited exposure to structured cardiovascular education may further contribute to this under-recognition. Additionally, public health campaigns that overemphasize atypical or sex-specific presentations might inadvertently weaken awareness of classic signs such as chest pain and dyspnea. Studies in both Western and Middle Eastern populations have shown that vague or overlapping symptoms often lead to delayed care-seeking, especially among women and older adults (11).

The second domain evaluated awareness of HA risk factors. Family history was the most recognized (RII = 70.99, medium level), followed by unhealthy diet (RII = 64.39), high cholesterol (RII = 63.92), diabetes (RII = 61.48), physical inactivity (RII = 59.98), past history of HA (RII = 57.70), and stress (RII = 57.15). While these reflect a moderate level of public understanding, awareness of smoking (RII = 43.71) and obesity (RII = 43.08) was notably poor, ranking last among all risk factors. This is concerning given their well-established role as modifiable contributors to CVD.

Several factors may explain this knowledge gap, including the cultural normalization of smoking and overweight status, insufficient health education, and stigma surrounding lifestyle-related risks. To address this, public health interventions must go beyond generic awareness campaigns. Strategies should incorporate locally relevant visuals, community testimonials, and religious leader engagement to foster cultural resonance. Health promotion efforts can be embedded in schools, workplaces, mosques, and primary care clinics to improve risk literacy and encourage behavior change. National media campaigns and social media initiatives should emphasize the dangers of smoking and obesity in simple, relatable formats. These tailored interventions could help bridge the gap between medical knowledge and public understanding, ultimately improving cardiovascular outcomes.

Table 4 shows the attitudes toward seeking medical care. ‘I would be embarrassed to go to the hospital if I thought I was having a heart attack, but I wasn’t’ has the highest RII (67.36, high level). ‘Because of the cost of medical care, I would want to be sure I was having a heart attack before going to the hospital’ ranks second (RII = 66.08, medium level). ‘If I thought I was having a heart attack, I would wait until I was very sure before going to the hospital’ ranks third (RII = 59.01, medium level).

**Table 4:** Attitudes toward seeking medical care.


ATTITUDES TOWARD SEEKING MEDICAL CARE	M	SD	RII	LA	R

‘If I have chest pain that doesn’t stop after 15 minutes, I should get to the hospital as soon as possible’	1.559	0.82589	31.18	Very low	7

‘If I thought I was having a heart attack, I would wait until I was very sure before going to the hospital’	2.9505	1.31365	59.01	Medium	3

‘If I thought I was having a heart attack, I would go to the hospital right away’	1.6156	0.91783	32.312	Very low	6

‘If I’m having chest pain and I’m not very sure if it’s a heart attack, I should go to the hospital’	2.092	1.07564	41.84	Low	5

‘I would be embarrassed to go to the hospital if I thought I was having a heart attack but I wasn’t’	3.3679	1.33564	67.358	High	1

‘Because of the cost of medical care, I would want to be absolutely sure I was having a heart attack before going to the hospital’	3.3042	1.33842	66.084	Medium	2

‘If I thought I was having a heart attack, I would rather have someone drive me to the hospital than have an ambulance come to my home’	2.3066	1.34405	46.132	Low	4


Abbreviations: LA, level of agreement; M, mean; R, rank; RII, relative importance index ((mean/3) × 100%); SD, standard deviation.

Items like ‘If I have chest pain that doesn’t stop after 15 minutes, I should get to the hospital as soon as possible’ (RII = 31.18, very low) and ‘If I thought I was having a heart attack, I would go to the hospital right away’ (RII = 32.31, very low) are among the lowest-ranked responses. The preference for self-transport over calling an ambulance is low (RII = 46.13, low level). The item ‘If I’m having chest pain and I’m not very sure if it’s a heart attack, I should go to the hospital’ is also low (RII = 41.84, low).

**Figure 2:**
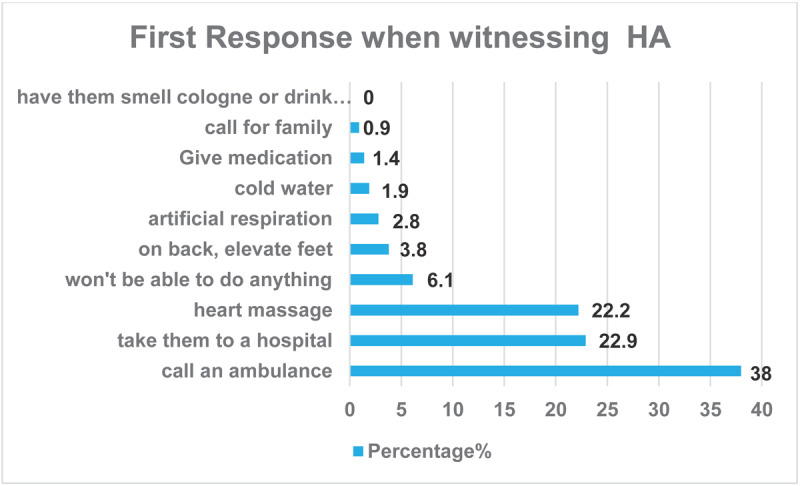
First response of the participants when witnessing heart attack (HA).

Figure 2 shows that 38% of respondents believed that the appropriate first step with an HA patient is to call an ambulance, followed by taking the person to a hospital (23%) and performing heart massage (22%). Notably, 6.1% of the participants stated that they would not be able to take any action in such a situation.

Table 5 provides percentages of participants across different demographics who correctly identified various symptoms related to HAs. A significant difference in awareness is observed across age groups; participants aged 46–55 years (28.7%) and 36–45 years (26.8%) had higher recognition of the ‘sudden pain in jaw, neck, or back’ symptom compared to the other age groups (*P* = 0.001). The 26–35 age group (36.6%) showed higher awareness of ‘sudden disturbance of vision in one or both eyes’. The 18–25 age group (33.8%) demonstrated the highest recognition of ‘numbness or tingling in hand’ (*P* = 0.032). However, no significant differences were observed between males and females in recognizing HA symptoms.

**Table 5:** The associations between demographics and heart attack awareness.


	PERCENTAGES OF THOSE WHO CORRECTLY IDENTIFIED WHETHER THE FOLLOWING ARE HA SYMPTOMS OR NOT									

	SUDDEN PAIN IN JAW, NECK, OR BACK	WEAKNESS OR DIZZINESS	SUDDEN PAIN IN THE CHEST	SUDDEN DISTURBANCE OF VISION IN ONE OR BOTH EYES	SUDDEN PAIN IN ARMS OR SHOULDERS	SUDDEN SHORTNESS OF BREATH	ABDOMINAL PAIN.	SLURRED SPEECH	NUMBNESS OR TINGLING IN THE HAND	PALPITATION

Age, years										

18–25	12.1	26	22.2	23.9	13	24.5	17.3	27.5	33.8	23.8

26–35	19.7	22.1	21.6	36.6	18.4	20.6	17.3	20.3	23.9	22

36–45	26.8	21	22.8	21.1	28.1	23.9	25.3	23.2	21.1	22.9

46–55	28.7	18.8	21.9	14.1	28.1	20.9	30.7	23.2	14.1	21.3

≥65	12.7	12.2	11.4	4.2	12.4	10.1	9.3	5.8	7	10.1

*P* value	0.001	0.149	0.35	0.011	0.001	0.498	0.4	0.442	0.032	0.114

Sex										

Female	51.6	53.6	54	52.1	54.1	52.6	50.7	42	43.7	53.4

Male	48.4	46.4	46	47.9	45.9	47.4	49.3	58	56.3	46.6

*P* value	0.899	0.682	0.102	0.179	0.574	0.393	0.555	0.149	0.374	0.246

Education level										

Illiterate	2.5	2.8	2.2	0	2.2	2	0	1.4	1.4	2.1

Primary school	1.3	1.1	1.2	0	1.6	1.3	2.7	0	0	0.9

Middle school	3.8	3.3	2.8	0	2.7	3.3	5.3	2.9	2.8	2.7

High school	19.7	26.5	24.7	38	21.1	25.5	18.7	31.9	32.4	27.1

Diploma	13.4	13.8	11.4	12.7	13	13.1	13.3	18.8	15.5	12.2

Bachelor’s degree	52.2	44.8	50.9	42.3	52.4	49.3	50.7	40.6	45.1	49.1

Postgraduate	7	7.7	6.8	7	7	5.6	9.3	4.3	2.8	5.8

*P* value	0.813	0.248	0.446	0.19	0.028	0.856	0.068	0.364	0.006	0.537

Marital status										

Single	14.6	24.3	21.6	35.2	13	23.9	21.3	33.3	36.6	23.5

Married	80.3	71.8	74.7	60.6	83.2	72.2	73.3	55.1	59.2	71.6

Divorced	1.9	2.2	2.2	4.2	1.6	2.3	4	7.2	4.2	3.4

Widow	3.2	1.7	1.5	0	2.2	1.6	1.3	4.3	0	1.5

*P* value	0.002	0.453	0.09	0.06	0.001	0.284	0.977	0.002	0.002	0.551

Employment status										

Employed	49	43.6	42.3	43.7	47.6	40.8	48	43.5	35.2	42.7

Unemployed	10.2	8.8	10.2	9.9	7	9.2	10.7	7.2	7	8.8

Student	10.8	21.5	18.8	23.9	11.4	20.9	14.7	26.1	33.8	20.7

House wife	13.4	14.9	17	15.5	20	17.6	8	14.5	14.1	17.7

Retired	16.6	11	11.7	7	14.1	11.4	18.7	8.7	9.9	10.1

*P* value	0.002	0.665	0.589	0.439	0.001	0.429	0.162	0.529	0.096	0.737


Abbreviation: HA, heart attack.

Awareness of the symptom ‘sudden pain in arms or shoulders’ was significantly associated with education level (*P* = 0.028), with participants holding a bachelor’s degree showing the highest percentage of awareness (52.4%). However, participants with high school (32.4%) and diploma (15.5%) showed higher awareness of ‘numbness or tingling in the hand symptom’ than other groups (*P* = 0.006).

There was a significant difference by marital status (*P* = 0.002), with married individuals (80.3%) showing higher recognition of ‘sudden pain in jaw, neck, or back’ symptoms. Additionally, the ‘numbness or tingling in the hand’ symptom has a significant variation (*P* = 0.002) with married participants (59.2%).

Forty-nine percent of employed participants had an awareness of ‘sudden pain in jaw, neck, or back’ symptoms with a significant difference (*P* = 0.002), and 47.6% of them knew ‘sudden pain in arms or shoulders’ symptoms with a significant awareness difference (*P* = 0.001).

Table 6 shows that no significant differences (*P* > 0.05) were found in symptom recognition based on pre-existing health conditions, including hypertension, diabetes, dyslipidemia, heart disease, or stroke.

**Table 6:** The associations between pre-existing health condition and heart attack awareness.


	PERCENTAGES OF THOSE WHO CORRECTLY IDENTIFIED WHETHER THE FOLLOWING ARE HA SYMPTOMS OR NOT									

	SUDDEN PAIN IN JAW, NECK, OR BACK	WEAKNESS OR DIZZINESS	SUDDEN PAIN IN THE CHEST	SUDDEN DISTURBANCE OF VISION IN ONE OR BOTH EYES	SUDDEN PAIN IN ARMS OR SHOULDERS	SUDDEN SHORTNESS OF BREATH	ABDOMINAL PAIN	SLURRED SPEECH	NUMBNESS OR TINGLING IN THE HAND	PALPITATION

Hypertension										

Yes	17.8	16	14.5	9.9	16.2	15.7	16	8.7	11.3	13.4

No	82.2	84	85.5	90.1	83.8	84.3	84	91.3	88.7	86.6

*P* value	0.177	0.119	0.301	0.488	0.427	0.253	0.751	0.314	0.281	0.348

DM										

Yes	14	10.5	11.1	7	12.4	11.1	16	8.7	8.5	11.3

No	86	89.5	88.9	93	87.6	88.9	84	91.3	91.5	88.7

*P* value	0.106	0.546	0.448	0.521	0.63	0.34	0.226	0.295	0.672	0.308

Dyslipidemia										

Yes	9.6	8.8	7.1	5.6	8.6	7.8	9.3	5.8	7	7.6

No	90.4	91.2	92.9	94.4	91.4	92.2	90.7	94.2	93	92.4

*P* value	0.234	0.358	0.929	0.814	0.399	0.293	0.612	0.032	0.04	0.457

Heart diseases										

Yes	1.3	3.3	3.4	1.4	3.2	3.6	1.3	5.8	1.4	3.7

No	98.7	96.7	96.6	98.6	96.8	96.4	98.7	94.2	98.6	96.3

*P* value	0.003	0.158	0.833	0.557	0.623	0.927	0.466	0.511	0.563	0.752

Stroke										

Yes	0	0	0.6	0	0.5	0.3	0	0	0	0.3

No	100	100	99.4	100	99.5	99.7	100	100	100	99.7

*P* value	0.127	0.246	0.733	0.815	0.762	0.643	0.792	0.785	0.349	0.53


Abbreviation: HA, heart attack.

## Discussion

Prompt recognition of HA symptoms is critical to reducing mortality, yet public awareness remains suboptimal, especially for classic signs such as chest pain and shortness of breath. Our findings mirror this trend, with these symptoms being among the least recognized despite their clinical importance. Similar gaps have been reported in Saudi and US populations, where less than half of respondents could identify all key HA symptoms. These deficits underline the need for targeted, culturally tailored awareness campaigns to improve timely care-seeking behavior (13).

Most respondents (63.8%) were aware of HA and the importance of immediate treatment; however, only 53.5% could correctly identify the appropriate ambulance emergency number. The majority of health information was obtained through the internet and social media, with supplementary input from healthcare professionals, television, and printed literature. Notably, direct engagement with healthcare professionals was reported by only 15.94% of participants, indicating a decreased reliance on expert consultations.

Supporting these findings, Abdo Ahmed et al. (14) identified a significant association between individuals informed about HA through family history, public service announcements, social media, and other digital channels, and increased awareness of HA symptoms and the need for urgent treatment. These results are consistent with similar studies conducted in the United States and South Korea (15,16), reinforcing the importance of trusted health communication. To counter misinformation, health authorities must prioritize the development of certified online educational resources.

A significant percentage of individuals demonstrated knowledge of prevalent HA symptoms, including stomach pain or abdominal pain (72.9%), weakness or dizziness (66.35%), and sudden disturbance of vision in one or both eyes (65.33%), indicating a higher identification of these symptoms relative to others. Nonetheless, recognition of other significant symptoms, such as dyspnea and discomfort in the jaw, neck, or back, was somewhat diminished. This restricted acknowledgment of some critical symptoms corresponds with prior research indicating that chest discomfort is often the most acknowledged indicator. In contrast, atypical symptoms are undervalued, especially among younger and less educated demographics. Our results aligned with the literature about the predominant symptoms of ACS (17,18), perhaps due to the widespread recognition of chest pain and discomfort as hallmark symptoms of ACS. This may be due to cultural misinterpretation of these symptoms as non-cardiac or minor.

Moreover, some individuals remain oblivious to the fact that heart disease may present with gastrointestinal symptoms. An in-hospital research indicates that the majority of patients identify the symptoms of ACS based on prior experience or acquaintance with individuals who have had an HA. Nonetheless, it is also shown that having a personal history of ACS does not ensure the recognition of all pertinent indicators and may influence their medical treatment (19). Consequently, the inability to inform the community and hospitalized patients about ACS symptoms may lead to a deficiency in knowledge. Collaborations with healthcare practitioners to augment digital visibility and boost information quality may be essential in disseminating precise heart health education and perhaps altering perceptions about timely medical intervention.

Regarding risk variables, participants demonstrated considerable knowledge of elements such as family history (70.99 RII) and elevated cholesterol levels (63.92 RII). However, modifiable risk factors like smoking and obesity exhibited lower levels of awareness. This mismatch illustrates a prevalent knowledge deficit seen in several countries, where lifestyle-related hazards may not be entirely acknowledged as manageable factors contributing to CVDs. Al Harbi et al. (20) identified obesity as the predominant risk factor for CVD, followed by smoking, cholesterol, and hypertension, with 47.7% of individuals diagnosed with diabetes. Prior research by Alwakeel et al. (21) revealed smoking as the predominant risk factor recognized by participants among the general population of the Tabuk area in Saudi Arabia. This indicates the need for targeted educational campaigns that emphasize the critical nature of lifestyle-related risks.

Furthermore, Alwakeel et al. (21) indicated that most patients do not acknowledge the connection between diabetes mellitus and ACS, aligning with our observations. Obesity and smoking are controllable risk factors; thus, increasing public knowledge of the MI risk linked to these behaviors may decrease its prevalence. This research indicates that several significant risk factors for HA, such as diabetes, are inadequately comprehended. Numerous prior research yielded similar results (22,23). Enhancing public awareness of the importance of these modifiable variables may diminish HA’s occurrence since behavioral modifications may directly influence cardiovascular health outcomes.

In terms of the attitudes toward pursuing medical treatment, the statement ‘I would feel embarrassed to visit the hospital if I believed I was experiencing a heart attack but was not’ has the highest RII at 67.36, indicating a significant degree of concern. ‘Due to the expense of medical treatment, I would want to be entirely certain I was experiencing an HA before seeking hospital care’ ranks second (RII = 66.08, medium level). ‘If I suspected a heart attack, I would wait until I was certain before seeking hospital care’ ranks third (RII = 59.01, medium level). This study indicates a prudent strategy whereby people may postpone seeking treatment until they ascertain the severity of their symptoms. This inclination may pose a danger, considering the urgent nature of HA intervention. Consistent with previous research, our participants’ opinions suggest they might struggle to recognize HA symptoms in others (17,24). Moreover, the mean overall attitude score was comparable to that documented by Dracup et al. (25) but inferior to that recorded by O’Brien et al. (17). These disparities might be ascribed to cultural variations.

Thirty-eight percent of respondents believed that the suitable first action for a patient experiencing an HA is calling an ambulance, transporting the individual to a hospital (23%), and administering cardiac compressions (22%). The findings indicate a strong general awareness of the need for emergency reaction during an HA, with contacting an ambulance being the favored course of action. Nevertheless, many responders either favor hospital transfer or cardiac massage, which may not consistently represent the most suitable urgent actions. Likewise, Abdo Ahmed et al. (14) indicated that 35.6% of participants deemed contacting an ambulance an appropriate response when an individual has an HA. Contrarily, studies conducted in other nations showed varied findings. In Poland (26), South Korea (15), and the United States (27), the percentages of respondents who saw contacting an ambulance as a suitable action were 87.4%, 67%, and 86%, respectively. These results emphasize the need for public education in identifying HA symptoms, the essential function of contacting emergency services, and the appropriate use of CPR to enhance preparedness and successful response. The research indicates reluctance and obstacles in pursuing prompt medical care during a suspected MI. A total of 6.1% of the participants said they would be unable to act in the case.

Furthermore, several respondents indicated they would delay visiting the hospital until they were certain they were experiencing HA, primarily due to concerns about shame (RII = 67.36) and healthcare expenses (RII = 66.08). Such attitudes can lead to dangerous delays in seeking care, as timely medical intervention is critical for favorable outcomes in HA cases.

The reluctance to call an ambulance—reflected in a preference for personal transportation to the hospital (RII = 46.13)—suggests a lack of awareness about the urgency of the condition and the potential life-saving care paramedics can provide en route. These findings highlight the urgent need for targeted public health initiatives to dispel misconceptions and emphasize the importance of immediate action when HA symptoms occur.

To address these barriers, public health campaigns should aim to destigmatize emergency care through culturally sensitive education, involving trusted community and religious leaders. Financial concerns could be alleviated by increasing public awareness of free emergency services or by incorporating emergency care costs into national health insurance programs. Additionally, mobile clinics and educational workshops in underserved areas can help reduce both psychological and financial hesitancy, reinforcing the life-saving value of timely medical attention.

Marked disparities (*P* < 0.05) in knowledge of specific HA symptoms were observed across various demographic factors, including age, education, marital status, and job status. Certain age groups, educational levels, and marital conditions demonstrated greater awareness of critical symptoms. However, findings by Al Harbi et al. (20) indicated that while age was not a statistically significant factor influencing HA knowledge overall, older individuals were less likely to have inadequate knowledge. Additionally, men showed a higher likelihood of having poorer knowledge compared to women. Although education level showed a positive correlation with HA knowledge, this relationship was not statistically significant (20).

This heterogeneity in awareness highlights the need for targeted educational interventions tailored to specific demographic groups. For instance, health promotion strategies for younger adults (18,19,20,21,22,23,24,25) could leverage short-form, entertaining educational videos on platforms such as TikTok and Instagram to enhance engagement and retention. In contrast, adults aged 45 years and older might respond better to information delivered through WhatsApp community groups, mosque-based health seminars, or local health campaigns.

For individuals with low literacy levels, communication through pictorial and audio-based content—distributed via public display screens, community centers, or local radio—can effectively convey essential health information. Furthermore, married individuals may benefit from couple-focused workshops or counseling sessions that emphasize mutual health responsibilities.

Implementing demographic-specific strategies ensures that HA awareness campaigns are not only inclusive but also optimized for maximum reach and effectiveness, ultimately helping to close the knowledge gaps across population subgroups.

### Strengths and limitations

This study has several notable strengths. It utilized a validated and reliable questionnaire, which enhanced the consistency and credibility of the data collected. The use of mandatory response settings in the online survey helped minimize missing data, resulting in a more complete and robust dataset. Importantly, the study addresses a critical public health issue: awareness of HA symptoms within the context of Al-Hasa, a region where such localized data are limited. As a result, the findings contribute valuable insight into the broader field of cardiovascular health in Saudi Arabia and similar settings.

Despite these strengths, the study also has several limitations. The cross-sectional design restricts the ability to draw causal inferences; while associations between demographic factors and HA awareness were observed, temporal relationships could not be established. The geographic focus on Al-Hasa may limit generalizability to other regions in Saudi Arabia. In addition, the exclusive reliance on online surveys may have excluded individuals without internet access, such as the elderly, low-income individuals, or those with limited digital literacy, potentially introducing sampling bias. Future studies should consider mixed-method approaches, including telephone or paper-based surveys, to ensure broader population coverage.

Furthermore, the self-reported nature of the data may have introduced recall bias and social desirability bias, particularly in responses related to prior exposure to HA education and symptom knowledge. Some participants may have overstated their awareness to align with perceived expectations. Non-response bias may also be present if participants differed systematically from those who chose not to respond, particularly in terms of their interest or awareness of health topics. Additionally, while illiterate participants were allowed to receive assistance in completing the questionnaire, this may have introduced interpretation bias. The presence of a helper, though well-intentioned, could have influenced how questions were understood or how responses were selected. To improve standardization in future research, interviewer-administered or audio-assisted formats may be more suitable for participants with limited literacy.

## Conclusion

Most of the population demonstrated moderate to good awareness of common symptoms and risk factors associated with HAs. However, the primary source of information for most respondents was the internet and social media, rather than healthcare professionals, which raises concerns about the accuracy and depth of the knowledge acquired. Despite relatively high awareness, the study revealed a poor attitude toward seeking medical care during an HA. Only half of the population could correctly identify the emergency ambulance number, and many participants expressed a tendency to delay care until they were certain of the symptom severity. Additionally, concerns about cost and embarrassment emerged as significant barriers to prompt care-seeking behavior. This is particularly alarming given the time-sensitive nature of HA treatment, where delays can lead to poor outcomes or even death.

These findings highlight a critical need for targeted public health strategies. Campaigns should aim to increase recognition of hallmark symptoms such as chest pain and shortness of breath by integrating content into school curricula, mass media, and digital platforms. Risk education must also explicitly address modifiable factors such as smoking and obesity, using culturally sensitive formats to overcome stigma and misinformation. The use of real-life testimonials, community health programs, and social media influencers can be particularly effective in promoting accurate knowledge and behavior change.

Policymakers should implement routine awareness programs in schools, workplaces, and religious centers while also expanding free public access to emergency care information. Together, these interventions can empower individuals to recognize early warning signs, seek immediate medical help, and ultimately reduce the burden of heart disease in the community.

## Additional Files

The additional files for this article can be found as follows:

10.5334/gh.1492.s1 Table (S1). Reliability of the survey using Cronbach’s alpha.

10.5334/gh.1492.s2 Table (S2). Validity test of the survey.

## Abbreviations

CVDs, cardiovascular diseases

HA, heart attack

ACS, acute coronary syndrome

MI, myocardial infarction

RII, relative importance index

CVI, content validity index

SPSS, Statistical Package for the Social Sciences
