# Supplementary material for: Public Awareness and Attitude Regarding the Symptoms of Heart Attacks
Source: Glob Heart. 2025 Dec 2;20(1):106. doi: 10.5334/gh.1492 (PMC12680000; doi:10.5334/gh.1492)
Supplement: Table (S1). — Reliability of the survey using Cronbach’s alpha. [file gh-20-1-1492-s1.pdf]

Table (S1) Reliability of the survey using Cronbach's alpha:

Domain	Cronbach alpha
First domain symptoms of HA	79.6
Second domain risk factors	82.4
The third domain attitudes towards seeking medical care	76.3
All domain	80.7

Table (1) shows the reliability of the survey. It was assessed using Cronbach's alpha, which measures internal consistency within each domain: Cronbach's alpha for the first domain is 0.796, indicating good reliability. This value suggests that items within the symptoms domain consistently measure similar aspects of awareness. For the second domain, Cronbach's alpha = 0.824, this domain also demonstrates strong reliability, showing high consistency in responses regarding risk factor knowledge. The third Domain, Cronbach's alpha, is 0.763, which still indicates acceptable reliability, though slightly lower than the other domains. This may reflect more attitude variability compared to symptom or risk factor awareness. The Cronbach's alpha for the entire survey is 0.807, showing robust reliability across all domains. Generally, an alpha above 0.7 is considered acceptable, so these results support the survey's reliability.
