# Supplementary material for: Public Awareness and Attitude Regarding the Symptoms of Heart Attacks
Source: Glob Heart. 2025 Dec 2;20(1):106. doi: 10.5334/gh.1492 (PMC12680000; doi:10.5334/gh.1492)
Supplement: Table (S2). — Validity test of the survey. [file gh-20-1-1492-s2.pdf]

Table (S2): Validity test of the survey.

First domain	validity	Second domain	validity	Third domain	validity
1	.682**	1	.535**	1	.676**
2	.410**	2	.572**	2	.607**
3	.500**	3	.585**	3	.575**
4	.562**	4	.591**	4	.632**
5	.558**	5	.626**	5	.603**
6	.518**	6	.798**	6	.727**
7	.775**	7	.566**	7	.563**
8	.579**	8	.438**		
9	.576**	9	.597**		
10	.640**	10	.622**		
11	.582**				

Table 2 shows the correlation of each item within each domain, with each item demonstrating a significant positive correlation (marked by **). These correlations reflect the construct validity of the items, indicating that each item within the domains effectively contributes to the overall measured construct (symptoms, risk factors, or attitudes).

In the first Domain (Symptoms of HA), the correlations range from 0.410 to 0.775, indicating varying but significant strengths of association among items. The highest correlation (0.775) suggests that this item strongly reflects awareness of symptoms, while the lowest (0.410) still contributes positively to the second Domain (Risk Factors). The correlations here range from 0.438

to 0.798, indicating that most items are moderately to strongly correlated within the domain. The highest correlation (0.798) implies a strong item association with the risk factor construct. In the third Domain (Attitudes toward Seeking Medical Care), Correlations range from 0.563 to 0.727, reflecting moderate to strong associations among items related to attitudes. These correlations confirm that items are effectively capturing the construct of attitudes toward seeking medical care.
